# Defect‐Free Few‐Layer M_4_C_3_T_x_ (M = V, Nb, Ta) MXene Nanosheets: Synthesis, Characterization, and Physicochemical Properties

**DOI:** 10.1002/advs.202302882

**Published:** 2023-08-02

**Authors:** Yanan Huang, Jibing Shen, Shuai Lin, Wenhai Song, Xuebin Zhu, Yuping Sun

**Affiliations:** ^1^ Key Laboratory of Materials Physics Institute of Solid State Physics HFIPS Chinese Academy of Sciences Hefei Anhui 230031 P. R. China; ^2^ University of Science and Technology of China Hefei 230026 P. R. China; ^3^ High Magnetic Field Laboratory HFIPS Chinese Academy of Sciences Hefei Anhui 230031 P. R. China; ^4^ Collaborative Innovation Centre of Advanced Microstructures Nanjing University Nanjing Jiangsu 210093 P. R. China

**Keywords:** defect‐free nanosheets, few‐layer M_4_C_3_T_x_ (M = V, Nb, Ta) MXenes, free‐standing film, intercalation and exfoliation, physicochemical properties, selective etching

## Abstract

High‐quality few‐layer M_4_C_3_T_x_ (M = V, Nb, Ta) MXenes are very important for applications and are necessary for clarifying their physicochemical properties. However, the difficulty in etching for themselves and the existence of MC/MC_1−δ_ and M‐Al alloy impurities in their M_4_AlC_3_ precursors seriously hinder the achievement of defect‐free few‐layer M_4_C_3_T_x_ (M = V, Nb, Ta) MXenes nanosheets. Herein, three different defect‐free few‐layer M_4_C_3_T_x_ (M = V, Nb, Ta) nanosheets are obtained by using a universal synthesis strategy of calcination, selective etching, intercalation, and exfoliation. Comprehensive characterizations confirm their defect‐free few‐layer structure feature, large interlayer spacing (1.702–1.955 nm), types of functional groups (–OH, –F, –O), and abundant valance states (M^5+^, M^4+^, M^3+^, M^2+^, M^0^). M_4_C_3_T_x_ (M = V, Nb, Ta) free‐standing films obtained by vacuum filtration of few‐layer M_4_C_3_T_x_ inks show good hydrophilia, high thermostability, and conductivity. A roadmap on synthesis of defect‐free few‐layer M_4_C_3_T_x_ (M = V, Nb, Ta) nanosheets are proposed and three key points are summarized. This work provides detailed guidelines for the synthesis of other defect‐free few‐layer MXenes nanosheets, but also will stimulate extensive functional explorations for M_4_C_3_T_x_ (M = V, Nb, Ta) MXenes nanosheets in the future.

## Graphical abstract

Three different defect‐free few‐layer M_4_C_3_T_x_ (M = V, Nb, Ta) MXenes nanosheets with large interlayer spacing, rich valance state, good flexibility, hydrophilic, and high conductivity and thermal stability have been successfully achieved via a universal synthesis strategy of high‐temperature calcination, selective etching, intercalation, and exfoliation. A roadmap and related attention points on synthesis of defect‐free few‐layer M_4_C_3_T_x_ nanosheets are proposed.

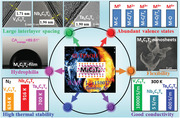

## 1 Introduction

Recently, two‐dimensional (2D) transition metal carbides and nitrides, known as MXenes, have attracted considerable attention.^[^ ^1^ ^]^ Thanks to their 2D structure, outstanding conductivity, good mechanical, and unique physiochemistry properties, MXenes have been extensively studied in many fields, such as energy storage, catalysis, sensing, biological medicine, electromagnetic shielding, and so on.^[^ ^2^, ^3^ ^]^ The general formula of MXene is M_n+1_X_n_T_x_ (*n* = 1–4), where M is the transitional metals, X is carbon/nitrogen, and T_x_ stands for surface functional groups, such as –O, –F, –OH, –Cl, and so on.^[^ ^2^ ^]^ As *n* value increasing, there are four different structure types of MXenes: M_2_CT_x_, M_3_C_2_T_x_, M_4_C_3_T_x_, and M_5_C_4_T_x_. So far, more than 40 kinds of MXenes have been experimentally synthesized and most researches are focused on M_2_CT_x_ and M_3_C_2_T_x_ systems.^[^ ^2^, ^3^ ^]^ M_4_C_3_T_x_ and M_5_C_4_T_x_ MXenes have been relatively less studied, which are difficult to synthesize.

As a kind of typical MXenes, M_4_C_3_T_x_ (M = V, Nb, Ta) MXenes have shown strong oxidation resistance^[^ ^4^ ^]^ and excellent electrochemical activity.^[^ ^5^, ^6^, ^7^, ^8^, ^9^ ^]^ For example, multi‐layer M_4_C_3_T_x_ (M = V, Nb, Ta) MXenes show good supercapacitors performance with specific capacity up to 550 F g^−1^ for Ta_4_C_3_T_x_^[^ ^9^ ^]^ and 230 F g^−1^ for V_4_C_3_T_x_.^[^ ^10^ ^]^ As anode material of Li‐ion battery, both V_4_C_3_T_x_ and Nb_4_C_3_T_x_ show superior electrochemical performance.^[^ ^11^, ^12^ ^]^ Furthermore, M_4_C_3_T_x_ (M = V, Nb, Ta) MXenes also present good performance in catalysis and biology.^[^ ^7^, ^13^, ^14^, ^15^ ^]^ Up to now, most of studies on M_4_C_3_T_x_ (M = V, Nb, Ta) are multi‐layer structure,^[^ ^5^, ^7^, ^8^, ^9^, ^10^, ^11^, ^12^, ^14^, ^15^ ^]^ which are because it is difficult to obtain high‐quality few‐layer M_4_C_3_T_x_ (M = V, Nb, Ta). And, the reasons are as follows: i) Pure‐phase M_4_AlC_3_ (M = V, Nb, Ta) precursors are difficult to obtain, and the impurities (MC/MC_1−δ_) will hinder the effect of selective etching; ii) The strong M─Al bonds in M_4_AlC_3_ (M = V, Nb, Ta) are difficult to completely etch under general etching conditions; iii) The kinds of intercalating agent and stripping process to achieve high efficient exfoliation without damaging M_4_C_3_ skeleton layers are not yet known. However, it is necessary to obtain few‐layer and even single‐layer M_4_C_3_T_x_ (M = V, Nb, Ta) MXenes to further improve their functional properties and clarify their inherent physicochemical properties.

Therefore, we propose an optimizing synthesis strategy including precursor calcination, HF etching, intercalation, and exfoliation processes to obtain high‐quality few‐layer M_4_C_3_T_x_ (M = V, Nb, Ta) MXenes. As a result, three different defect‐free few‐layer M_4_C_3_T_x_ (M = V, Nb, Ta) MXenes with large interlayer spacing and rich valance state have been successfully achieved, which are confirmed by a series of characterizations. Furthermore, we obtain M_4_C_3_T_x_ (M = V, Nb, Ta) free‐standing films by vacuum filtration of “M_4_C_3_T_x_ (M = V, Nb, Ta) inks” and study their physicochemical properties such as hydrophilia, thermostability, and electrical conductivity. Considering the features of large interlayer spacing, abundant valance states, good hydrophilia, high thermostability, and electrical conductivity of few‐layer M_4_C_3_T_x_ (M = V, Nb, Ta) MXenes, it will result in extensive application explorations in the future.

## 2 Results and Discussion

**Figure 1:**
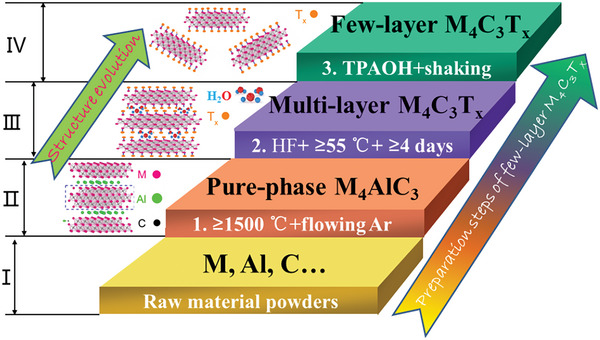
The preparation steps of few‐layer M_4_C_3_T_x_ (M = V, Nb, Ta) MXenes and structure evolution from 3D M_4_AlC_3_ to 2D few‐layer M_4_C_3_T_x_ (M = V, Nb, Ta) MXenes.

**Figure** **1** shows the schematic diagram of synthesis process of few‐layer M_4_C_3_T_x_ (M = V, Nb, Ta) MXene nanosheets. The key reactants and synthesis conditions have been labeled on the diagram. At the same time, the schematic diagram of crystal structure from 3D M_4_AlC_3_ to 2D few‐layer M_4_C_3_T_x_ (M = V, Nb, Ta) MXenes are also presented in Figure 1. According to the above steps, a series of M_4_AlC_3_ (M = V, Nb, Ta) MAX phases, multi‐layer as well as few‐layer M_4_C_3_T_x_ (M = V, Nb, Ta) MXenes were prepared. The detailed synthesis processes are listed in the part of Experimental Section.

**Figure 2:**
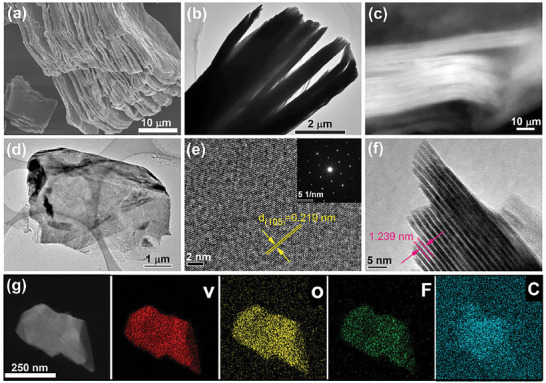
a) SEM image of multi‐layer V_4_C_3_T_x_; TEM bright field image b) and dark field image c) of out‐of‐plane multi‐layer V_4_C_3_T_x_; d) TEM image of in‐plane multi‐layer V_4_C_3_T_x_; e) HRTEM images of in‐plane multi‐layer V_4_C_3_T_x_ and the inset shows the corresponding SAED pattern; f) HRTEM image of out‐of‐plane multi‐layer V_4_C_3_T_x_; g) TEM‐EDX elemental mapping images of multi‐layer V_4_C_3_T_x_ MXene.

As a result, single‐phase V_4_AlC_3_ MAX phase was successfully obtained, which have been reported in our previous work.^[^ ^12^ ^]^ After HF etching, as shown in **Figure** **2**a–c, fully etched multi‐layer V_4_C_3_T_x_ MXenes with the microscopic appearance of accordion, which is a typical characteristic of MXene, were obtained. Here, it is worth noting that the accordion morphology of our multi‐layer V_4_C_3_T_x_ MXene is not a case but a universal existence (Figure S1, Supporting Information). SEM‐EDX results of multi‐layer V_4_C_3_T_x_ MXene show that Al layers are completely etched away (Figure S2, Supporting Information). Figure 2d presents the TEM image of muti‐layerV_4_C_3_T_x_ MXene. The in‐plane high‐resolution TEM (HRTEM) image of multi‐layer V_4_C_3_T_x_ is displayed in Figure 2e, from where we find that our as‐prepared multi‐layer V_4_C_3_T_x_ MXenes have an intact surface and there is no obvious defects or oxidation. As shown in Figure 2e and Figure S3a (Supporting Information), the d_(105)_ value of multi‐layer V_4_C_3_T_x_ MXene is 0.219 nm, which is consistence with that of V_4_AlC_3_ precursor.^[^ ^12^, ^16^ ^]^ The selected area electron diffractions (SAED) pattern of multi‐layer V_4_C_3_T_x_ MXene (the inset of Figure 2e) confirms the hexagonal structure and the in‐plane lattice constant *a* is 0.295 nm, which is agreement with the previous XRD results.^[^ ^12^, ^16^ ^]^ Figure 2f shows the cross‐section of multi‐layer V_4_C_3_T_x_ MXene with more than 10 layers, and the d_(002)_ value is 1.239 nm (Figure S3b, Supporting Information). Furthermore, TEM‐EDX results of multi‐layer V_4_C_3_T_x_ MXene show that there are only V, O, F, and C elements and they are evenly distributed (Figure 2g). The Al element cannot be detected in our as‐prepared multi‐layer V_4_C_3_T_x_ MXene, indicating the Al layers of V_4_AlC_3_ are completely etched. And, like other previously reported V_4_C_3_T_x_ MXene etched by HF,^[^ ^7^, ^10^, ^12^, ^15^, ^17^ ^]^ the surface functional groups of F, O, and/or OH are formed on V_4_C_3_ skeleton layers.

**Figure 3:**
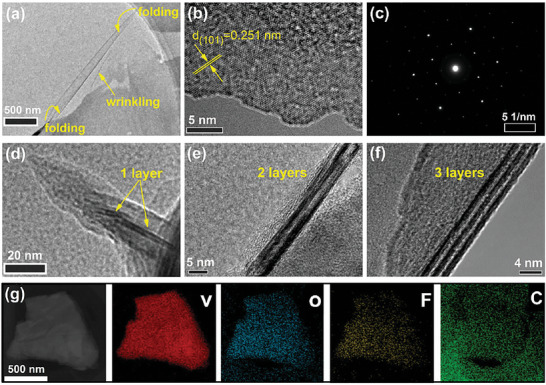
a) TEM image of few‐layer V_4_C_3_T_x_; b) HRTEM images of surface few‐layer V_4_C_3_T_x_ and c) corresponding SAED pattern; d–f) HRTEM images of cross‐sectional few‐layer V_4_C_3_T_x_ with different layers; g) TEM‐EDX elemental mapping images of few‐layer V_4_C_3_T_x_.

Few‐layer V_4_C_3_T_x_ was delaminated with tetrapropylammonium hydroxide (TPAOH) intercalation and vibration by hand. As shown in **Figure** **3**a, the TEM image of few‐layer V_4_C_3_T_x_ presents good flexibility with wrinkling and folding characterizations. The in‐plane HRTEM image of few‐layer V_4_C_3_T_x_ in Figure 3b displays an intact surface and its clear SAED pattern (Figure 3c) shows good hexagonal symmetry, indicating V_4_C_3_ skeleton layer are defect‐free with no hole and oxidation. That is to say, the intercalation and exfoliation processes do not damage the surface of few‐layer V_4_C_3_T_x_ while significantly reduce the number of layers (Figure 3d–f). At the same time, the in‐plane lattice constant (*a* = 0.294 nm) of few‐layer V_4_C_3_T_x_ is calculated from Figure 3c, which is almost equal to these of V_4_AlC_3_ and multi‐layer V_4_C_3_T_x_. In addition, as presented in Figure 3g, there are only V, O, F, and C elements for few‐layer V_4_C_3_T_x_ and they are evenly distributed, which are like these in multi‐layer V_4_C_3_T_x_.

**Figure 4:**
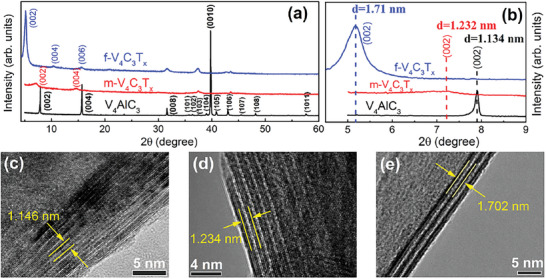
a,b) Room‐temperature XRD patterns of V_4_AlC_3_ and multi‐/few‐layer V_4_C_3_T_x_ (m‐/f‐V_4_C_3_T_x_); HRTEM images of cross‐sectional c) V_4_AlC_3_, d) m‐V_4_C_3_T_x_, and e) f‐V_4_C_3_T_x_, respectively.

**Figure** **4**a,b shows the XRD patterns of V_4_AlC_3_, multi‐layer V_4_C_3_T_x_, and few‐layer V_4_C_3_T_x_. As displayed in Figure 4a, the V_4_AlC_3_ is pure phase and all the diffraction peaks are consistent with theoretical calculation results.^[^ ^16^ ^]^ After HF etching, most of diffraction peaks corresponding to the V_4_AlC_3_ were either completely disappeared or greatly diminished in intensity, and the (00*l*) peaks broaden and downshift to low angles. After TPAOH intercalation and exfoliation, for few‐layer V_4_C_3_T_x_, there are only (00*l*) peaks and they further downshift to more low angles. Especially, as shown in Figure 4b, the interlayer spacing, namely, d_(002)_ value increases from 1.134 nm for V_4_AlC_3_ to 1.232 nm for multi‐layer V_4_C_3_T_x_, and even to 1.71 nm for few‐layer V_4_C_3_T_x_ with a net increasement of 0.576 nm. The enlarged interlayer spacings are also detected by HRTEM results, which are presented in Figure 4c–f and Figure S4 (Supporting Information). As mentioned above, we have confirmed that defect‐free few‐layer V_4_C_3_T_x_ MXene with large interlayer spacing have been successfully prepared by our proposed synthesis strategy.

**Figure 5:**
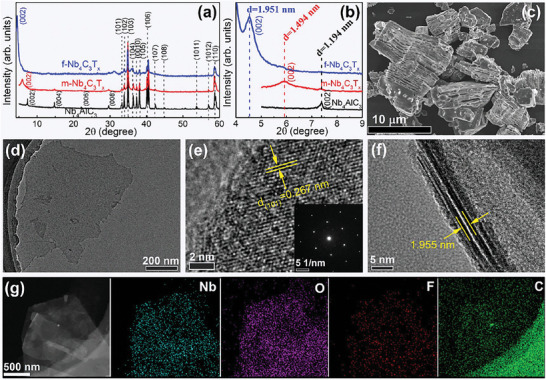
a,b) Room‐temperature XRD patterns of Nb_4_AlC_3_ and multi‐/few‐layer Nb_4_C_3_T_x_ (m‐/f‐Nb_4_C_3_T_x_); c) SEM image of m‐Nb_4_C_3_T_x_; d) TEM image of f‐Nb_4_C_3_T_x_; e) In‐plane HRTEM images of f‐Nb_4_C_3_T_x_ and the inset shows the corresponding SAED pattern; f) Cross‐sectional HRTEM images of f‐Nb_4_C_3_T_x_; g) TEM‐EDX elemental mapping images of f‐Nb_4_C_3_T_x_.

According to the synthesis strategy presented in Figure 1, we also synthesize defect‐free multi‐/few‐layer Nb_4_C_3_T_x_ and their single‐phase Nb_4_AlC_3_ precursor, which are confirmed by a series of XRD, SEM, EDX, and TEM characterization methods (see below). **Figure** **5**a shows the XRD patterns of our as‐synthesized Nb_4_AlC_3_ and multi‐/few‐layer Nb_4_C_3_T_x_. The Nb_4_AlC_3_ is single phase without obvious impurity and its SEM image displays layered structure features (Figure S5a, Supporting Information). After HF etching, for multi‐layer Nb_4_C_3_T_x_, the (002) peak broadens and moves to a lower angle (Figure 5b) and the accordion morphology is widely observed in Figure 5c and Figure S5b (Supporting Information), indicating a successful etching. And, the SEM‐EDX results of multi‐layer Nb_4_C_3_T_x_ MXene show that Al layers are almost completely etched away (Figure S6, Supporting Information). Further, after TPAOH intercalation and exfoliation, the in‐plane surface of as‐obtained few‐layer Nb_4_C_3_T_x_ is intact according to the SEM (Figure S5c, Supporting Information), TEM (Figure 5d), and HRTEM (Figure 5e) images. From the SAED pattern displayed in the inset of Figure 5e, we can obtain the lattice constant *a* = 0.314 nm for few‐layer Nb_4_C_3_T_x_, which is almost equal to that of its precursor Nb_4_AlC_3_ (*a* = 0.3129 nm).^[^ ^18^ ^]^ The d_(101)_ value of few‐layer Nb_4_C_3_T_x_ is 0.267 nm (Figure 5e and Figure S7a, Supporting Information), which is the same value as its Nb_4_AlC_3_ precursor.^[^ ^18^ ^]^ From the results of XRD (Figure 5b) and HRTEM images (Figure 5f, Figures S5d and S7b, Supporting Information), the interlayer spacing increases from 1.194 nm for Nb_4_AlC_3_ to 1.494 nm for multi‐layer Nb_4_C_3_T_x_, and even to 1.955 nm for few‐layer Nb_4_C_3_T_x_ with a max net increasement up to 0.761 nm. This will provide a large enough space for the transport and storage of various ions. Moreover, as presented in Figure 5g, there are only Nb, O, F, and C elements for few‐layer Nb_4_C_3_T_x_ and they are evenly distributed.

**Figure 6:**
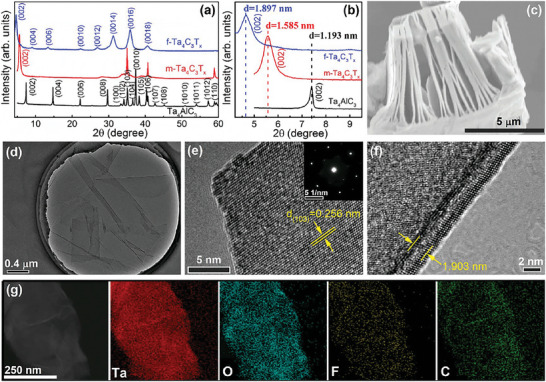
a,b) Room‐temperature XRD patterns of Ta_4_AlC_3_ and multi‐/few‐layer Ta_4_C_3_T_x_ (m‐/f‐Ta_4_C_3_T_x_); c) SEM image of m‐Ta_4_C_3_T_x_; d) TEM image of f‐Ta_4_C_3_T_x_; e) In‐plane HRTEM images of f‐Ta_4_C_3_T_x_ and the inset shows the corresponding SAED pattern; f) Out‐of‐plane HRTEM images of f‐Ta_4_C_3_T_x_; g) TEM‐EDX elemental mapping images of f‐Ta_4_C_3_T_x_.

Similarly, we also obtained pure‐phase Ta_4_AlC_3_ and defect‐free multi‐/few‐layer Ta_4_C_3_T_x_ (**Figure** **6**). To be more specific, as shown in Figure 6a, there is no obvious impurity peaks in the XRD diffraction peaks of Ta_4_AlC_3_ and the SEM image of Ta_4_AlC_3_ display a dense ladder of layered structure (Figure S8, Supporting Information). After HF etching, the (002) peak of multi‐layer Ta_4_C_3_T_x_ broadens and shifts to low angles (Figure 6b). The SEM‐EDX results of multi‐layer Ta_4_C_3_T_x_ MXene show that Al layers are completely etched away (Figure S9, Supporting Information). At the same time, obvious accordance morphology is found in Figure 6c, Figures S9a and S10a (Supporting Information). The in‐plane and out‐of‐plane HR‐TEM images of multi‐layer Ta_4_C_3_T_x_ show that the interlayer spacing increase while surface keep intact (Figure S10b–d, Supporting Information). After TPAOH intercalation and exfoliation, the (002) peak of few‐layer Ta_4_C_3_T_x_ downshifts to a lower angle, resulting in a large interlayer spacing of 1.897 nm with a max net increasement of 0.704 nm (Figure 6b). The size of our synthesized few‐layer Ta_4_C_3_T_x_ nanosheets is up to micrometer (Figure 6d) and display a good flexibility (Figure S11a, Supporting Information). As shown in Figure 6e, the in‐plane surface of few‐layer Ta_4_C_3_T_x_ is intact without any defects, impurities, and oxides. The d_(103)_ value of few‐layer Ta_4_C_3_T_x_ is 0.256 nm (Figure 6e), which is consistent with that of Ta_4_AlC_3_ precursor.^[^ ^19^ ^]^ As presented in Figure 6f, the interlayer spacing of few‐layer Ta_4_C_3_T_x_ is 1.903 nm, which is much close to the value obtained by XRD measurement (Figure 6b). Especially, few‐layer Ta_4_C_3_T_x_ were widely observed in Figure 6f and Figure S11b (Supporting Information), suggesting that our intercalation and exfoliation processes are adequate and efficient. Moreover, as displayed in Figure 6g, there are only Ta, O, F, and C elements for few‐layer Ta_4_C_3_T_x_ and they are evenly distributed, suggesting that the types of functional group are –O, –OH, and –F. In addition, the Fourier transform infrared spectroscopy (FTIR) measurements of few‐layer M_4_C_3_T_x_ (M = V, Nb, Ta) MXenes are carried out to further determine the type of their functional groups. As presented in Figure S12 (Supporting Information), the M–O, M–F, and –OH were observed in few‐layer M_4_C_3_T_x_ (M = V, Nb, Ta) MXenes, which are consistent with the results of multi‐layer M_4_C_3_T_x_ (M = V, Nb, Ta) MXenes reported previously.^[^ ^13^, ^20^, ^21^ ^]^ This suggests that the functional groups for few‐layer M_4_C_3_T_x_ (M = V, Nb, Ta) are mainly –OH, –O, and –F, which is consistent with the EDX results above.

**Figure 7:**
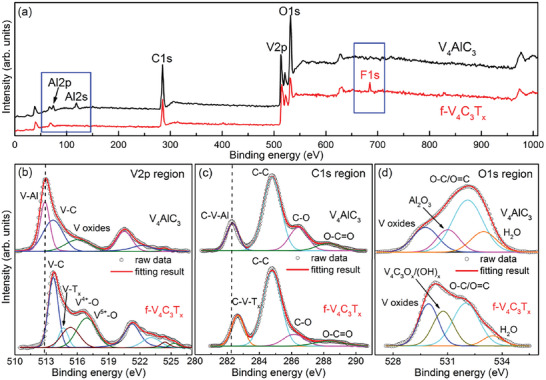
a) XPS spectra of V_4_AlC_3_ and few‐layer V_4_C_3_T_x_; High‐resolution XPS spectra of V_4_AlC_3_ and few‐layer V_4_C_3_T_x_ for b) V 2p, c) C 1s, and d) O 1s, respectively.

To further confirm the element composition, valence state, and the types of functional groups, the X‐ray photoelectron spectroscopy (XPS) measurements were performed on our samples. **Figure** **7** presents the XPS spectrum of few‐layer V_4_C_3_T_x_ MXene and its precursor V_4_AlC_3_. It is observed that the F 1s related peak centered at 684.9 eV is detected in few‐layer V_4_C_3_T_x_ rather than in V_4_AlC_3_ (Figure 7a), indicating the existence of F‐related termination in few‐layer V_4_C_3_T_x_. On the contrary, the Al 2p and Al 2s peaks disappear in few‐layer V_4_C_3_T_x_, confirming Al layers have been completely etched.

The high‐resolution spectrum of the V 2p region for the V_4_AlC_3_ and few‐layer V_4_C_3_T_x_ MXene are displayed in Figure 7b. For V_4_AlC_3_, the V2p region can be well fitted by the chemical bonds of V─Al (512.9 eV), V─C (513.7 eV), and V─O (516.9 eV) (in the top plot of Figure 7b).^[^ ^12^, ^17^, ^22^ ^]^ The V─O bond is resulted from the small amount of V oxides on the surface of V_4_AlC_3_.^[^ ^23^ ^]^ Considering the intermetallic compound feature of V_4_AlC_3_ (strong covalent and ionic bonds within the V_4_C_3_ layers, while weak metallic bonds between the V and Al layers), the V─Al (512.9 eV) and V─C (513.7 eV) bonds are related to the valance states of V^0^, V^2+^, and V^3+^.^[^ ^12^ ^]^ For few‐layer V_4_C_3_T_x_, Al layers were completely etched and V─Al bond was replaced by V─T_x_ bond. This leads the binding energy (BE) of V─T_x_ (514.7 eV) species shifting to a higher BE when compared to that of V─Al (512.9 eV) in V_4_AlC_3_ precursor. Importantly, the V 2p region of few‐layer V_4_C_3_T_x_ can be well fitted by several components including V^δ+^ (0 ≤*δ* ≤3) (V─C and V─T_x_ bonds of V_4_C_3_T_x_), V^4+^ (V─T_x_ bonds of V_4_C_3_T_x_ and V‐based oxides), and V^5+^ (V‐based oxides), showing abundant valence state.^[^ ^12^, ^20^, ^22^ ^]^

The high‐resolution spectrum in the C 1s region for V_4_AlC_3_ precursor can be well fitted by the following chemical bonds: C–V‐Al, C–C, C–O, and COO– (top plot of Figure 7c). The BE peak at 282.2 eV corresponds to the C─V─Al bond in V_4_AlC_3_.^[^ ^22^ ^]^ The BE peaks at 284.7, 286.4, and 288.3 eV are attributed to C─C, C─O, and O─C═O bonds, respectively.^[^ ^12^, ^20^, ^22^ ^]^ For few‐layer V_4_C_3_T_x_, C 1s region can be well fitted by C–V–T_x_ (282.6 eV), C–C, C–O, and COO– (bottom plot of Figure 7c). Obviously, the BE of C–V–T_x_ (282.6 eV) is higher than that of C–V–Al (282.2 eV), which is in line with V 2p region (Figure 7b).

The spectrum in the O 1s region (Figure 7d) for V_4_AlC_3_ can be well fitted by vanadium oxides (V─O bond) (529.8 eV), aluminum oxide (Al─O bond) (531 eV), C–O/C = O (532.1 eV), and physical adsorption of H_2_O (533.1 eV), while for few‐layer V_4_C_3_T_x_ MXene the O 1s region was fitted by following components: vanadium oxides (V─O bond) (529.8 eV), V_4_C_3_T_x_ (T_x_ = O_x_/OH_x_) (530.7 eV), C–O/C = O (532.1 eV), and physical and chemical adsorption of H_2_O (533.4 eV). From the XPS spectra, the few‐layer V_4_C_3_T_x_ MXene is terminated by a type of F, O, and/or OH species together with the presence of physical/chemical adsorption of H_2_O, which is like what were found in other MXenes.^[^ ^22^, ^24^ ^]^ In addition, the XPS spectra of few‐layer V_4_C_3_T_x_ is identical to that of multi‐layer V_4_C_3_T_x_ (Figure S13, Supporting Information), suggesting that the intercalation and exfoliation processes do not affect the valence states and compositions of V_4_C_3_T_x_ MXene.

**Figure 8:**
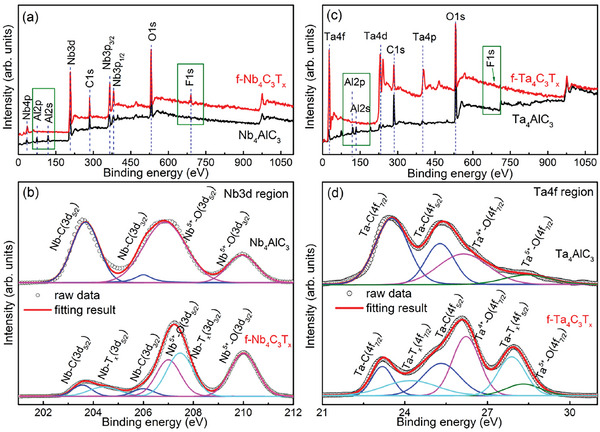
XPS spectra of Nb_4_AlC_3_ as well as few‐layer a) Nb_4_C_3_T_x_ and corresponding high‐resolution XPS spectra for b) Nb 3d; XPS spectra of Ta_4_AlC_3_ and few‐layer c) Ta_4_C_3_T_x_ and corresponding high‐resolution XPS spectra for d) Ta 4f.

Similarly, XPS measurements for Nb_4_AlC_3_ and few‐layer Nb_4_C_3_T_x_ as well as Ta_4_AlC_3_ and few‐layer Ta _4_C_3_T_x_ MXene were also carried out (**Figure** **8**). As displayed in Figure 8a,c, F peaks appear while Al 2p and 2s peaks disappear for few‐layer Nb_4_C_3_T_x_ and Ta _4_C_3_T_x_ MXenes, indicating Al layers were completed etched and F functional groups were existed. The high‐resolution spectrum in the Nb 3d region of Nb_4_AlC_3_ can be well fitted by the Nb–C (203.6 eV for Nb 3d_5/2_, 206 eV for Nb 3d_3/2_) and Nb–O (207 eV for Nb 3d_5/2_, 210 eV for Nb 3d_3/2_) (top plot of Figure 8b), while Nb 3d region of Nb_4_C_3_T_x_ can be well fitted by the Nb–C (203.6 eV for Nb 3d_5/2_, 206 eV for Nb 3d_3/2_), Nb–T_x_ (204.4 eV for Nb 3d_5/2_, 207.4 eV for Nb 3d_3/2_), Nb–O (207 eV for Nb 3d_5/2_, 210 eV for Nb 3d_3/2_) (bottom plot of Figure 8b).^[^ ^25^, ^26^, ^27^ ^]^ Correspondingly, the valance states of Nb are Nb^δ+^ (0 ≤ *δ* ≤ 3) for Nb─C and Nb─T_x_ bonds as well as Nb^4+^ and Nb^5+^ for Nb─O bonds, displaying a characteristic of polyvalence state (Nb^0^, Nb^2+^, Nb^3+^, Nb^4+^, and Nb^5+^). Similarly, as for high‐resolution spectra of Ta 4f region (Figure 8d), Ta_4_AlC_3_ and few‐layer Ta_4_C_3_T_x_ both have the Ta─C (23.3 eV for Ta 4f_7/2_, 25.3 eV for Ta 4f_5/2_) and Ta─O (Ta^4+^ at 26.1 eV for Ta 4f_7/2_ and Ta^5+^ at 28.3 eV for Ta 4f_7/2_) bonds.^[^ ^9^, ^13^ ^]^ However, Ta─T_x_ bond (including Ta^2+^ and Ta^3+^) (24.2 eV for Ta 4f_7/2_, 27.8 eV for Ta 4f_5/2_) is necessary to fit well the peaks in Ta 4f region of few‐layer Ta_4_C_3_T_x_ MXene,^[^ ^13^ ^]^ suggesting that Ta possesses the polyvalence state (Ta^0^, Ta^2+^, Ta^3+^, Ta^4+^, and Ta^5+^).

**Figure 9:**
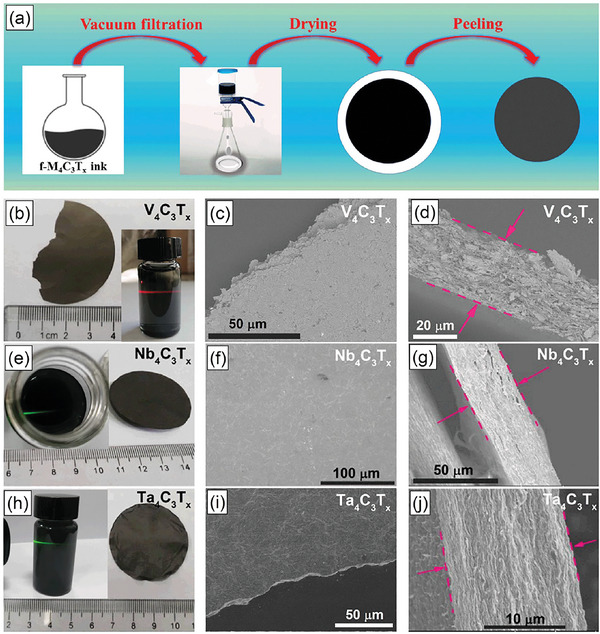
a) The diagram of preparation process of f‐M_4_C_3_T_x_ (M = V, Nb, Ta) free‐standing films. Photograph, surface and cross‐section SEM images for b–d) V_4_C_3_T_x_, e–g) Nb_4_C_3_T_x_, h–j) Ta_4_C_3_T_x_ free‐standing films, respectively. The insets of figures (b), (e), (h) show the few‐layer V_4_C_3_T_x_, Nb_4_C_3_T_x_, and Ta_4_C_3_T_x_ aqueous solutions, respectively.

As described above, we have obtained free‐defect few‐layer M_4_C_3_T_x_ (M = V, Nb, Ta) MXenes nanosheets and their structure, micromorphology, and valence state are characterized in detail. To further clarify their basic physicochemical properties such as conductivity, hydrophilicity, thermal stability, we obtain the few‐layer M_4_C_3_T_x_ (M = V, Nb, Ta) MXenes free‐standing films by vacuum filtration. **Figure** **9**a displays the diagram of M_4_C_3_T_x_ (M = V, Nb, Ta) free‐standing films preparation process. According to the above process, we successfully get V_4_C_3_T_x_ (Figure 9b–d), Nb_4_C_3_T_x_ (Figure 9e–g), Ta_4_C_3_T_x_ (Figure 9h–j) three different free‐standing films. Few‐layer M_4_C_3_T_x_ (M = V, Nb, Ta) nanosheets aqueous solution (namely, f‐M_4_C_3_T_x_ inks) show obvious Tyndall effect (the insets of Figure 9b,e,h)^[^ ^28^ ^]^, which are similar to other few‐layer MXene aqueous solutions such as Ti_3_C_2_T_x_, Ti_3_CNT_x_, V_2_CT_x_, Ti_2_C_0.5_N_0.5_T_x_ inks.^[^ ^29^, ^30^, ^31^, ^32^ ^]^ The surface SEM images of few‐layer M_4_C_3_T_x_ (M = V, Nb, Ta) films present dense feature, while the cross‐section SEM images display stacking effect. Furthermore, as shown in Figure S14 (Supporting Information), the free‐standing M_4_C_3_T_x_ (M = V, Nb, Ta) MXene films have a certain degree of flexibility and the M, O, C, and F elements are uniform distribution.

**Figure 10:**
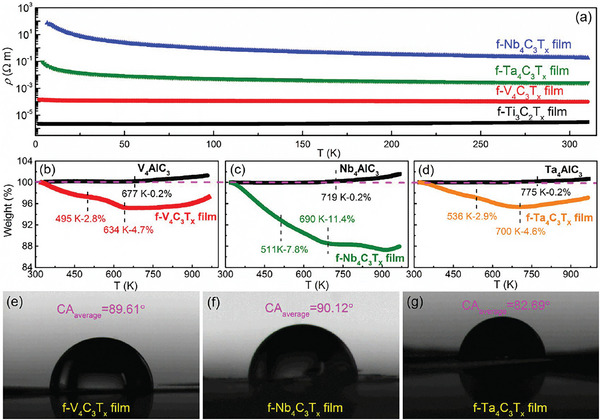
a) Temperature dependent resistivity of few‐layer M_4_C_3_T_x_ (M = V, Nb, Ta) and Ti_3_C_2_T_x_ free‐standing films. Thermogravimetric curves for few‐layer M_4_C_3_T_x_ (M = V, Nb, Ta) free‐standing films from room temperature to 1000 K in N_2_ atmosphere with heating rate of 10 K min^−1^: b) V_4_C_3_T_x_, c) Nb_4_C_3_T_x_, and d) Ta_4_C_3_T_x_. Photographs of the water droplet shape with the contact angle on few‐layer free‐standing films: e) V_4_C_3_T_x_, f) Nb_4_C_3_T_x_, and g) Ta_4_C_3_T_x_.

**Figure** **10**a, Figures S15, and S16 (Supporting Information) show the temperature dependence of resistivity for few‐layer M_4_C_3_T_x_ (M = V, Nb, Ta) and Ti_3_C_2_T_x_ free‐standing films. Different from the metallic behavior of Ti_3_C_2_T_x_ film, the resistivity of M_4_C_3_T_x_ (M = V, Nb, Ta) free‐standing films increases with decreasing temperature, showing a behavior of semiconductor.^[^ ^33^ ^]^ And, all the conductivities of M_4_C_3_T_x_ (M = V, Nb, Ta) are smaller than that of Ti_3_C_2_T_x_ throughout the whole test temperature range (Figure 10a). To be more precise, the room‐temperature electrical conductivity is 3.5 × 10^5^ S m^−1^ for Ti_3_C_2_T_x_, 1 × 10^4^ S m^−1^ for V_4_C_3_T_x_, 5 S m^−1^ for Nb_4_C_3_T_x_, and 400 S m^−1^ for Ta_4_C_3_T_x_, respectively.

Figure 10b‐d shows thermal gravimetry (TG) curves of M_4_AlC_3_ (M = V, Nb, Ta) and few‐layer M_4_C_3_T_x_ (M = V, Nb, Ta) free‐standing films from room temperature to 1000 K in N_2_ atmosphere. In the case of V_4_C_3_T_x_, the first stage of mass loss about 2.81% can be observed in the temperature range of 300–495 K, which is due to the removal of physically absorbed water and intercalated water;^[^ ^34^ ^]^ Between 495 and 634 K, the second stage of mass loss is 4.75%, which is resulted from the removal of functional groups.^[^ ^34^ ^]^ Above 634 K, the mass of sample increases with increasing temperature, indicating few‐layer V_4_C_3_T_x_ reacts with N_2_ atmosphere and was converted to other materials. At this point, the thermal stability of few‐layer V_4_C_3_T_x_ breakdown. Similarly, as presented in Figure 10c,d, the TG curves of few‐layer Nb_4_C_3_T_x_ and Ta_4_C_3_T_x_ display the same evolvement rule as that of few‐layer V_4_C_3_T_x_. And the thermal stability temperature is up to 690 K for few‐layer Nb_4_C_3_T_x_ and 700 K for few‐layer Ta_4_C_3_T_x_. For M_4_AlC_3_ (M = V, Nb, Ta) MAX phase, we define the temperature where the mass begins to increase (>0.2%) as the thermal instability temperature. The thermal stability temperatures of M_4_AlC_3_ (M = V, Nb, Ta) are high up to 677 K for V_4_AlC_3_, 719 K for Nb_4_AlC_3_, and 775 K for Ta_4_AlC_3_. It is found that the thermal stability temperatures of few‐layer M_4_C_3_T_x_ (M = V, Nb, Ta) MXenes are not much lower than those of their precursors. Our obtained free‐defect few‐layer M_4_C_3_T_x_ (M = V, Nb, Ta) have higher thermal stability temperature than those of other MXenes such as V_2_CT_x_, Ti_3_C_2_T_x_.^[^ ^35^, ^36^ ^]^ This is mainly due to the structure stability and free‐defect features of M_4_C_3_T_x_ (M = V, Nb, Ta).^[^ ^5^, ^37^ ^]^ In addition, as shown in Figure 10e–g, the few‐layer M_4_C_3_T_x_ (M = V, Nb, Ta) free‐standing films show a certain hydrophilicity with the contact angle around 90°. This will benefit their applications in water‐related energy storage, catalysis, and sensing.

**Figure 11:**
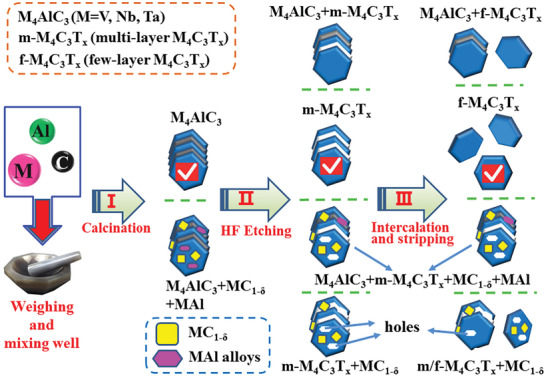
Roadmap on synthesis of defect‐free few‐layer M_4_C_3_T_x_ (M = V, Nb, Ta) MXenes nanosheets.

Finally, as shown in **Figure** **11**, a roadmap on synthesis of defect‐free few‐layer M_4_C_3_T_x_ (M = V, Nb, Ta) nanosheets is summarized. Firstly, single‐phase M_4_AlC_3_ (M = V, Nb, Ta) MAX phase precursor is important and necessary for synthesis of defect‐free few‐layer M_4_C_3_T_x_ (M = V, Nb, Ta) nanosheets. If the MAl alloys and MC/MC_1‐δ_ impurities existence in M_4_AlC_3_ (M = V, Nb, Ta) precursor, they will hinder the achievement of defect‐free few‐layer M_4_C_3_T_x_ (M = V, Nb, Ta). Specially speaking, the MAl alloys will lead to holes after HF etching, while the MC/MC_1−δ_ impurities will hinder the completed HF etching. Secondly, completed HF etching is required. If Al layers were not completely etched, it will affect the intercalation effect of next step. That is to say, the unetched Al will bond with upper and lower M_4_C_3_ (M = V, Nb, Ta) layers and hinder intercalation effect. Thirdly, suitable intercalation agent (TPAOH) as well as exfoliation process (shaking with hand) are very important for obtaining free‐defect few‐layer M_4_C_3_T_x_ (M = V, Nb, Ta). Excessive intercalation and stripping will destroy the M_4_C_3_ (M = V, Nb, Ta) skeleton layers, resulting in fragmentation or introduction of defects. Therefore, only when the above three steps are done well can we obtain free‐defect few‐layer M_4_C_3_T_x_ (M = V, Nb, Ta) MXene nanosheets. Namely, the route marked by the ticks is the optimal route to get high‐quality few‐layer M_4_C_3_T_x_ (M = V, Nb, Ta) MXenes.

## 3 Conclusions

In summary, we propose a universal synthesis strategy including high‐temperature calcination, HF etching, and intercalation exfoliation methods to solve the problem that high‐quality few‐layer M_4_C_3_T_x_ (M = V, Nb, Ta) MXenes are difficult to obtain. According to the above scheme, defect‐free few‐layer V_4_C_3_T_x_, Nb_4_C_3_T_x_, and Ta_4_C_3_T_x_ nanosheets, which are systematically confirmed by XRD, FTIR, XPS, SEM, TEM, and EDX, were successfully obtained. Three types of defect‐free M_4_C_3_T_x_ (M = V, Nb, Ta) nanosheets possessed few‐layer structure feature, flexibility, large interlayer spacing, and abundant valance states. M_4_C_3_T_x_ (M = V, Nb, Ta) free‐standing films obtained by vacuum filtration of few‐layer M_4_C_3_T_x_ (M = V, Nb, Ta) inks show hydrophilia, high thermostability and conductivity. Finally, three key suggestions are summarized to get defect‐free few‐layer M_4_C_3_T_x_ (M = V, Nb, Ta) MXene nanosheets. The achievement of defect‐free few‐layer M_4_C_3_T_x_ (M = V, Nb, Ta) nanosheets will stimulate people to carry out comprehensive and in‐depth application researches (such as oil adsorption,^[^ ^38^ ^]^ electromagnetic interference shielding,^[^ ^39^ ^]^ microwave absorption^[^ ^40^ ^]^) on M_4_C_3_T_x_ (M = V, Nb, Ta) MXenes in the future.

## 4 Experimental Section

### Material Synthesis

Polycrystalline MAX phase V_4_AlC_3_, Nb_4_AlC_3_, Ta_4_AlC_3_ were prepared by the conventional solid‐state reaction. Powders of vanadium (Aladdin, 99.5%, >325 mesh)/niobium (Aladdin, 99.9%, −325 mesh)/tantalum (Alfa Aesar, 99.5%, −325 mesh), aluminum (Aladdin, 99.9%, −200 mesh), and graphite (Sinopharm, 99.99%, −325 mesh) were mixed in a molar ratio of 4: 1.5: 2.8. After mixing well, the mixtures were pressed into disks under a pressure of 500 MPa and then heated in a tube furnace to 1500 °C for V_4_AlC_3_, 1600 °C for Nb_4_AlC_3_, and 1550 °C for Ta_4_AlC_3_, respectively. The heating rate was 9 °C min^−1^ between room temperature to 1000 °C and 5 °C min^−1^ from 1000 °C to final target temperatures under a flowing argon gas (>100 sccm). The samples were then annealed at target temperatures for 2 h before furnace cooling. After cooling to room temperature, the pellets of the sample were crumbled and sieved, and the obtained powders with grain size <38 µm were ready for further etching.

To prepare multi‐layer M_4_C_3_T_x_ (M = V, Nb, Ta) (m‐M_4_C_3_T_x_) MXenes, 1 g M_4_AlC_3_ (M = V, Nb, Ta) powders obtained above were slowly poured into a Teflon jar containing 20 mL hydrofluoric acid (HF) (Aladdin, 49 wt% in H_2_O) with continuous stirring at 500 rpm. And then, the reaction Teflon jar was put into an oil‐bath pan and stirred 96 h at 55 °C for V_4_C_3_T_x_, 100 h at 57 °C for Nb_4_C_3_T_x_, 110 h at 60 °C for Ta_4_C_3_T_x_, respectively. After HF treatment, the resulting suspensions were washed several times using deionized water (DI) until the pH value of supernatants was approximately neutral (pH > 5). The settled powders were dried in vacuum oven at 50 °C for 12 h.

Few‐layer M_4_C_3_T_x_ (M = V, Nb, Ta) (f‐M_4_C_3_T_x_) nanosheets were obtained by intercalation and exfoliation method. For 0.5 g of m‐M_4_C_3_T_x_ MXenes, 10 mL of TPAOH (Aladdin, 2.0 m in H_2_O) was used as the intercalation agent. The intercalated m‐M_4_C_3_T_x_ was stirred at 600 rpm for 18 h at 15 °C for V_4_C_3_T_x_ (higher temperatures such as 25 or 30 °C will lead to the oxidations and pores on the obtained V_4_C_3_T_x_ nanosheets), 30 °C for Nb_4_C_3_T_x_, and 35 °C for Ta_4_C_3_T_x_, respectively. After that, the intercalated powders were centrifuged (10 000 rpm, 10 min) and washed with DI water to remove excess TPAOH (pH < 8). And then, 100 mL of DI water was added and shook with hand for 15 min. The supernatant of delaminated flakes was collected by centrifugation (3000 rpm, 30 min). Free‐standing f‐M_4_C_3_T_x_ films were made via vacuum filtration on a porous cellulose membrane with pore size of 0.22 µm. And then, the free‐standing films can be peeled off from cellulose membrane after drying at room temperature.

### Material Characterizations

For characterizations, the X‐ray powder diffraction (XRD) was performed using a Philips X′pert PRO X‐ray diffractometer with Cu *K_α_* radiation (*λ* = 0.15 406 nm) at room temperature. The micro‐topographies and the compositions of as‐synthesized samples were determined by Field emission scanning electron microscope (FE‐SEM, Quanta 200 FEG) with Energy‐dispersive X‐ray spectroscopy (EDX, Oxford EDX, with INCA software), transmission electron microscope (TEM, JEM‐2100) with EDX, respectively. Fourier transform infrared spectroscopy (FTIR) was tested by NEXUS (Thermo Nicolet Corporation, America). X‐ray photoelectron spectroscopy (XPS) was measured by Thermo ESCALAB 250Xi equipped with monochromatic Al Ka source of 1486.6 eV.

### Physicochemical Property Measurements

Contact angle measurements of water were performed at room temperature using the sessile drop technique. The water drop was placed on the surface of f‐M_4_C_3_T_x_ (M = V, Nb, Ta) free‐standing films and the contact angles were measured from photographs taken with a CCD camera. The electrical transport properties of f‐M_4_C_3_T_x_ (M = V, Nb, Ta) free‐standing films were measured on a Quantum Design physical property measurement system (PPMS). The electrical transport properties were performed by a standard four probe setup (Figure S16, Supporting Information), where electrical contacts were made by using silver epoxy, to eliminate contact resistance for these low resistivity materials. Thermogravimetry (TG) was carried out on a thermal analyzer (STA, 449 F3, Netzsch, Germany) in N_2_ atmosphere at a heating rate of 10 °C min^−1^ from room temperature to 1000 K.

## Conflict of Interest

The authors declare no conflict of interest.

## Supporting information

Supporting Information

## Data Availability

The data that support the findings of this study are available from the corresponding author upon reasonable request.
