# Supplementary material for: Defect‐Free Few‐Layer M4C3Tx (M = V, Nb, Ta) MXene Nanosheets: Synthesis, Characterization, and Physicochemical Properties
Source: Adv Sci (Weinh). 2023 Aug 2;10(28):2302882. doi: 10.1002/advs.202302882 (PMC10558640; doi:10.1002/advs.202302882)
Supplement: Supplementary file 1 — Supporting Information [file ADVS-10-2302882-s001.pdf]

ADVANCED SCIENCE
Open Access

Supporting Information

for *Adv. Sci.*, DOI 10.1002/advs.202302882

Defect-Free Few-Layer $M_4C_3T_x$ (M = V, Nb, Ta) MXene Nanosheets: Synthesis, Characterization, and Physicochemical Properties

Yanan Huang, Jibing Shen, Shuai Lin, Wenhai Song, Xuebin Zhu* and Yuping Sun*

Supporting Information:

Defect-free few-layer $M_4C_3T_x$ (M=V, Nb, Ta) MXene nanosheets: Synthesis, characterization, and physicochemical properties

Yanan Huang[1#], Jibin Shen[1,2#], Shuai Lin[1*], Wenhai Song[1], Xuebin Zhu[1*], and Yuping Sun[1,3,4]

[1]Key Laboratory of Materials Physics, Institute of Solid State Physics, HFIPS, Chinese Academy of Sciences, Hefei, Anhui, 230031, People's Republic of China.
[2]University of Science and Technology of China, Hefei 230026, People’s Republic of China
[3]High Magnetic Field Laboratory, HFIPS, Chinese Academy of Sciences, Hefei, Anhui 230031, People's Republic of China.
[4]Collaborative Innovation Centre of Advanced Microstructures, Nanjing University, Nanjing, Jiangsu 210093, People's Republic of China.

*Corresponding authors and e-mail: linshuai17@issp.ac.cn and xbzhu@issp.ac.cn
[#]These authors contributed equally to this work.

Keywords: Few-layer $M_4C_3T_x$ (M=V, Nb, Ta) MXenes; Defect-free nanosheets; Selective etching; Intercalation and exfoliation; Free-standing film; Physicochemical properties

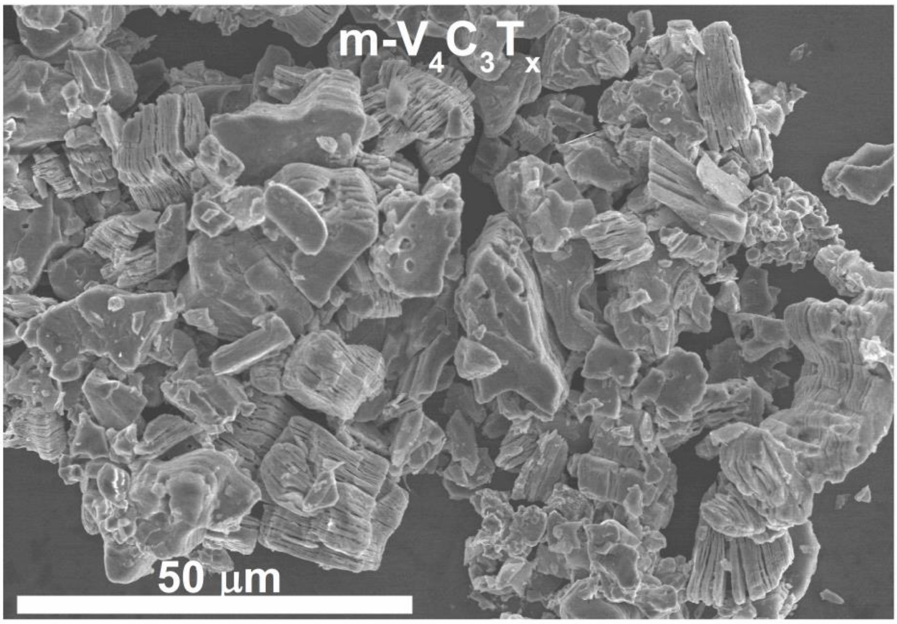

Fig. S1 SEM image of multi-layer $V_4C_3T_x$ (m-$V_4C_3T_x$).

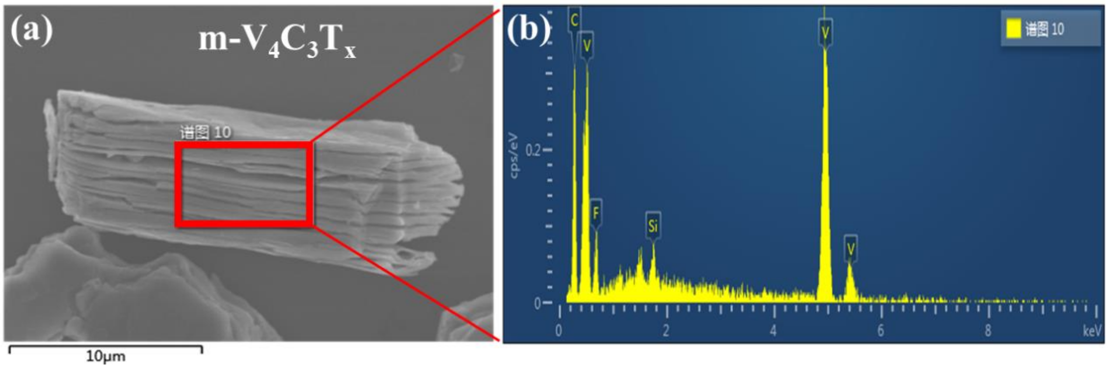

Fig. S2 SEM image (a) and EDX spectra (b) of multi-layer $V_4C_3T_x$ (m-$V_4C_3T_x$). The silicon element comes from the silicon wafer, on which m-$V_4C_3T_x$ samples are glued.

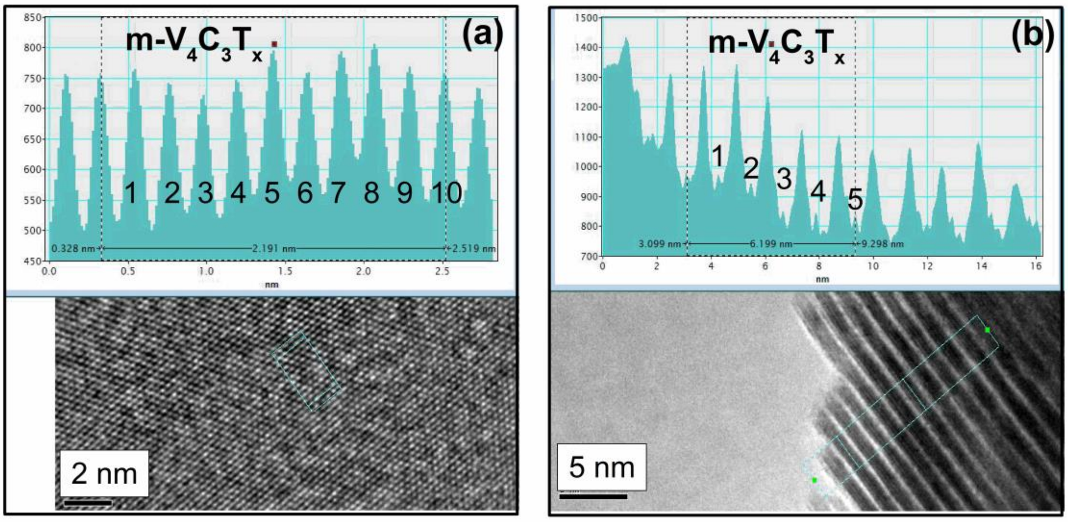

Fig. S3 TEM images of multi-layer $V_4C_3T_x$ (m-$V_4C_3T_x$) in-plane **(a)** and out-of-plane **(b)**, and corresponding fringe spacing measurements: **(a)** $d_{(105)}$ = 0.2191 nm; **(b)** $d_{(002)}$ = 1.24 nm.

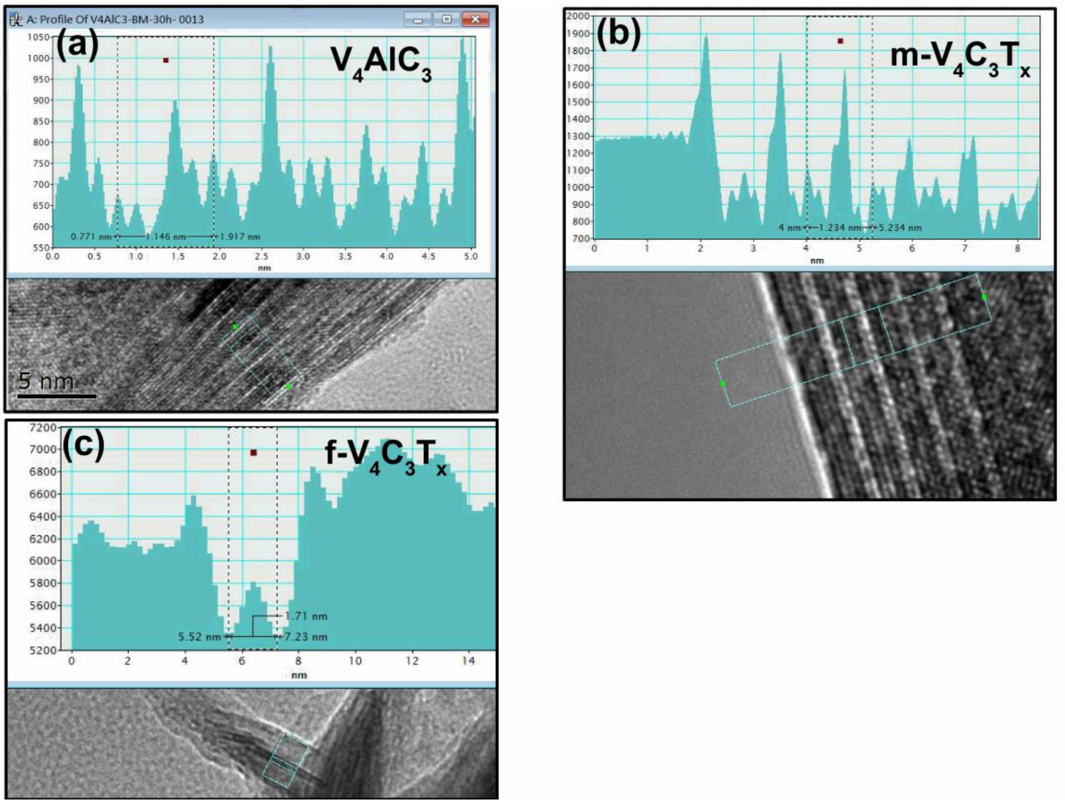

Fig. S4 TEM images of cross-section for V_4AlC_3 **(a)**, multi-layer $V_4C_3T_x$ (m-$V_4C_3T_x$) **(b)**, and few-layer $V_4C_3T_x$ (f-$V_4C_3T_x$) **(c)**, respectively, and corresponding fringe spacing measurements.

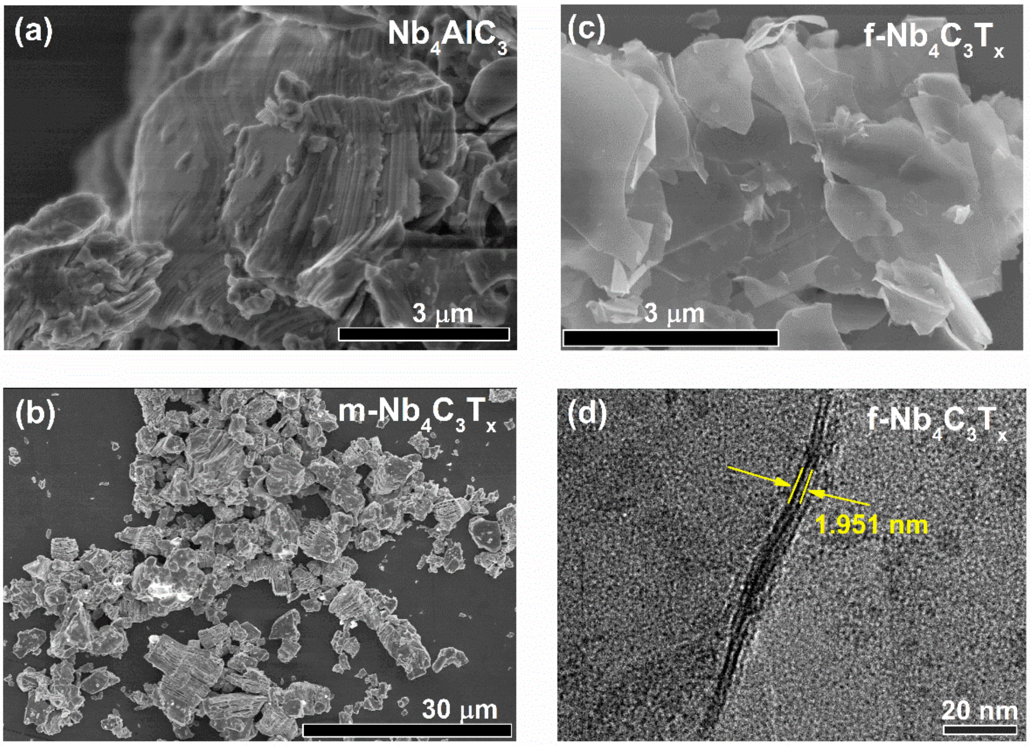

Fig. S5 SEM images of Nb_4AlC_3 **(a)**, multi-layer $Nb_4C_3T_x$ (m-$Nb_4C_3T_x$) **(b)**, and few-layer $Nb_4C_3T_x$ (f-$Nb_4C_3T_x$) **(c)**, respectively; **(d)** TEM image of few-layer $Nb_4C_3T_x$ (f-$Nb_4C_3T_x$).

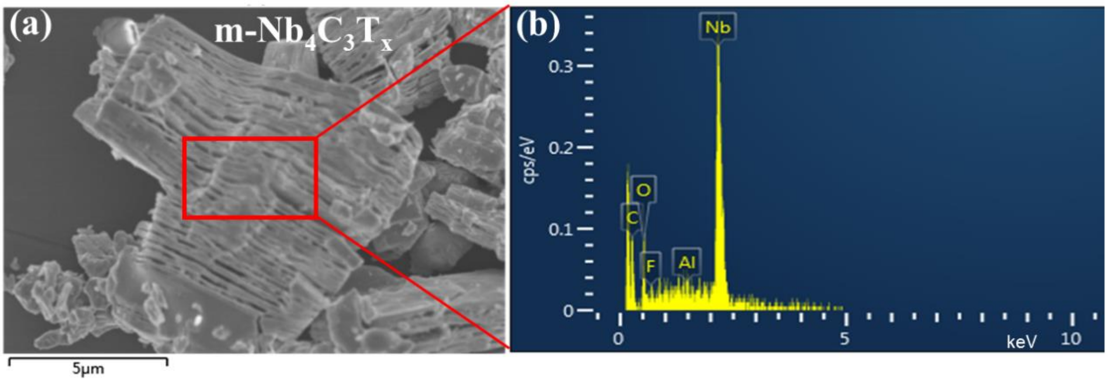

Fig. S6 SEM image (a) and EDX spectra (b) of multi-layer $Nb_4C_3T_x$ (m-$Nb_4C_3T_x$).

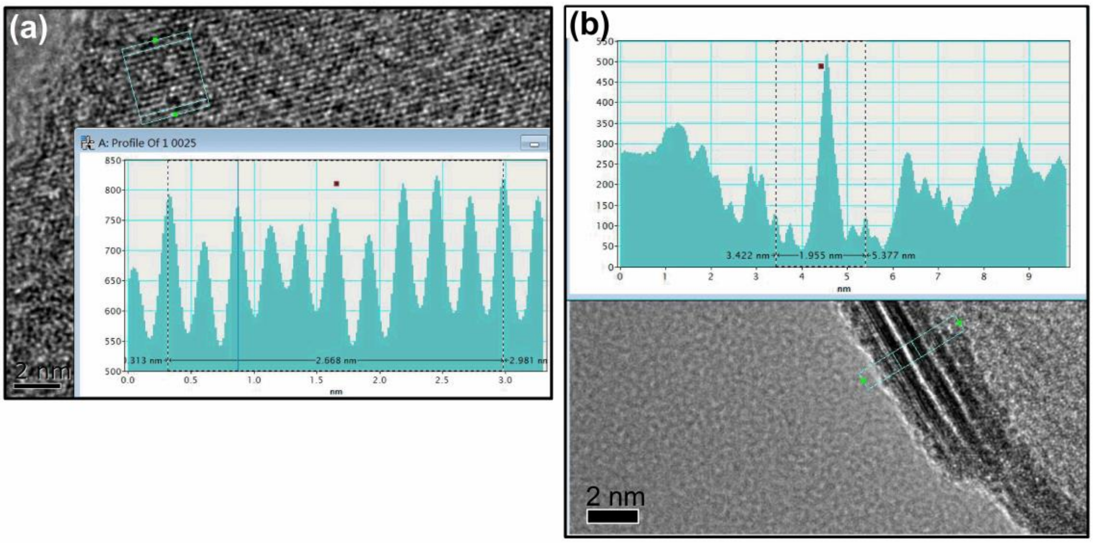

Fig. S7 The in-plane **(a)** and out-of-plane **(b)** TEM images of few-layer $Nb_4C_3T_x$ (f-$Nb_4C_3T_x$), and corresponding fringe spacing measurements.

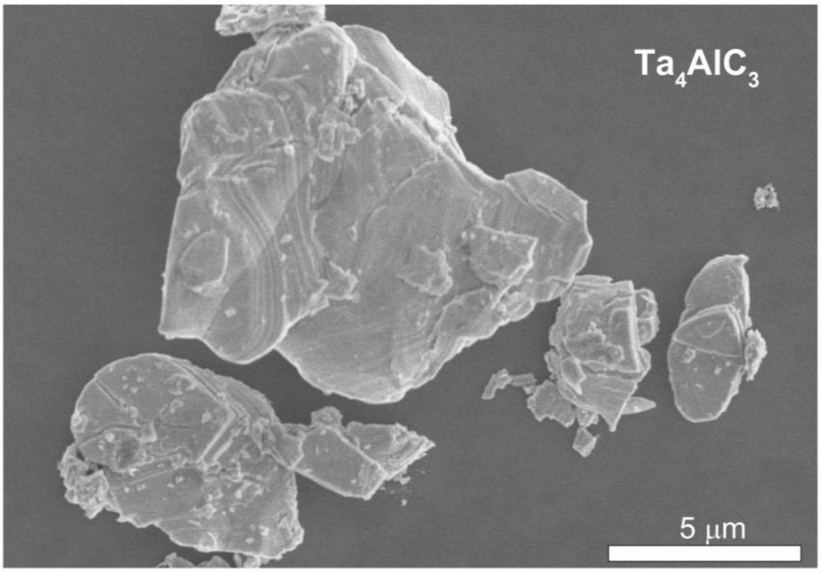

Fig. S8 SEM image of Ta_4AlC_3.

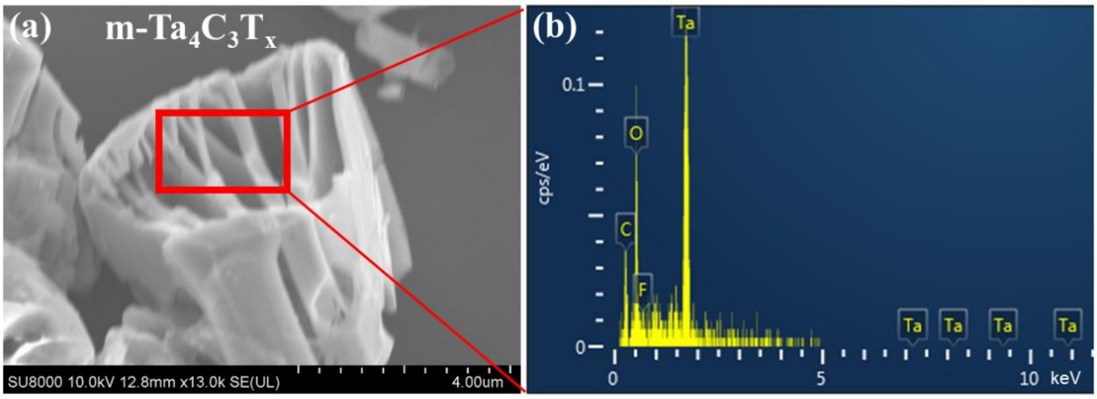

Fig. S9 SEM image (a) and EDX spectra (b) of multi-layer $Ta_4C_3T_x$ (m-$Ta_4C_3T_x$).

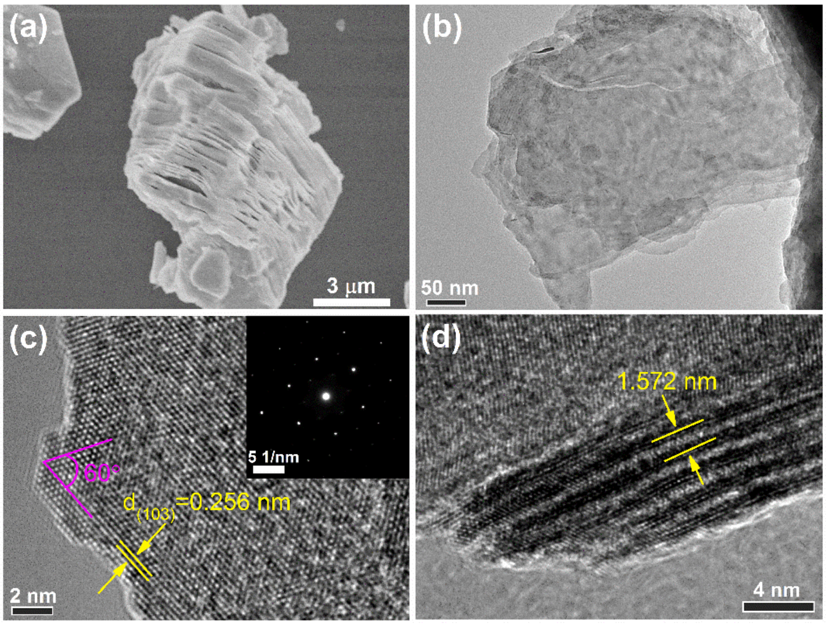

Fig. S10 (a) SEM image of multi-layer $Ta_4C_3T_x$ (m-$Ta_4C_3T_x$); **(b)** TEM image of m-$Ta_4C_3T_x$; **(c)** In-plane HRTEM image of m-$Ta_4C_3T_x$, and the inset show the corresponding SAED pattern; **(d)** Out-of-plane HRTEM image of m-$Ta_4C_3T_x$.

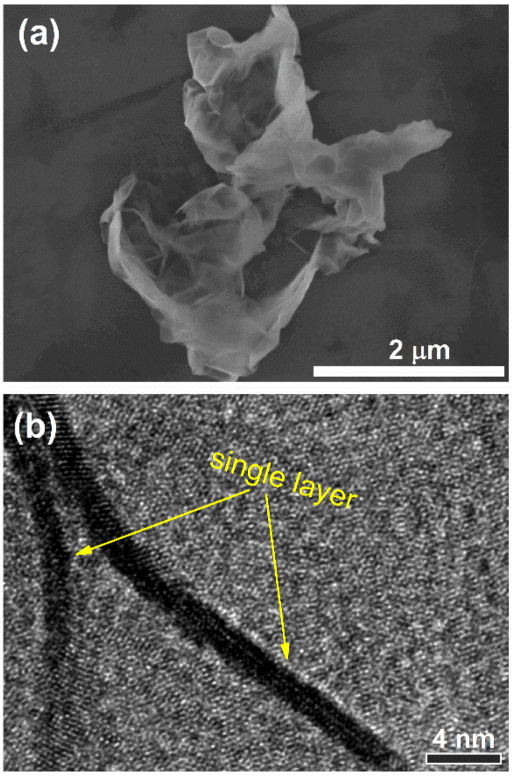

Fig. S11 (a) SEM image of few-layer $Ta_4C_3T_x$ (f-$Ta_4C_3T_x$); **(b)** HRTEM images of f-$Ta_4C_3T_x$.

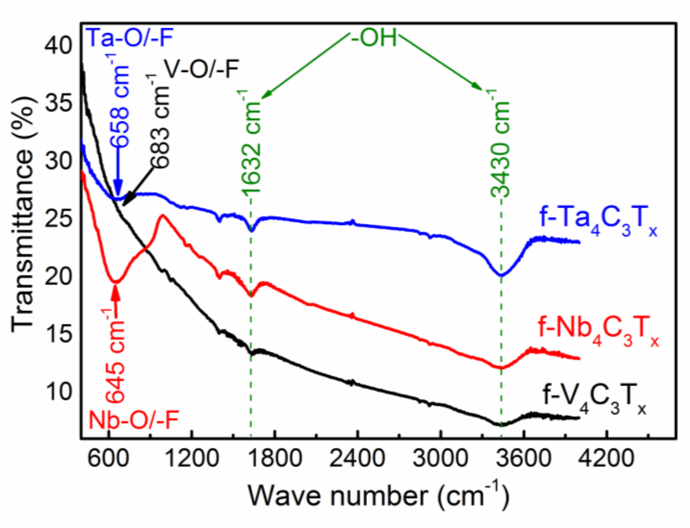

Fig. S12 Fourier transform infrared spectroscopy (FTIR) of few-layer $M_4C_3T_x$ (M=V, Nb, Ta) MXenes.

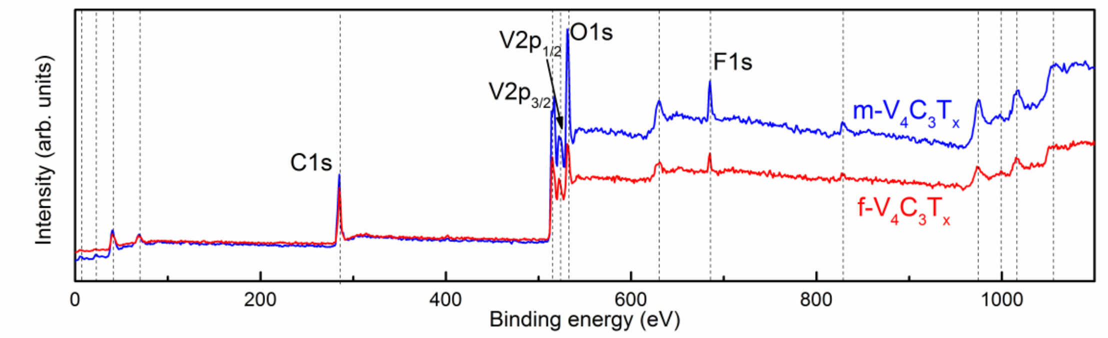

Fig. S13 XPS spectra of multi-layer $V_4C_3T_x$ (m-$V_4C_3T_x$) and few-layer $V_4C_3T_x$ (f-$V_4C_3T_x$).

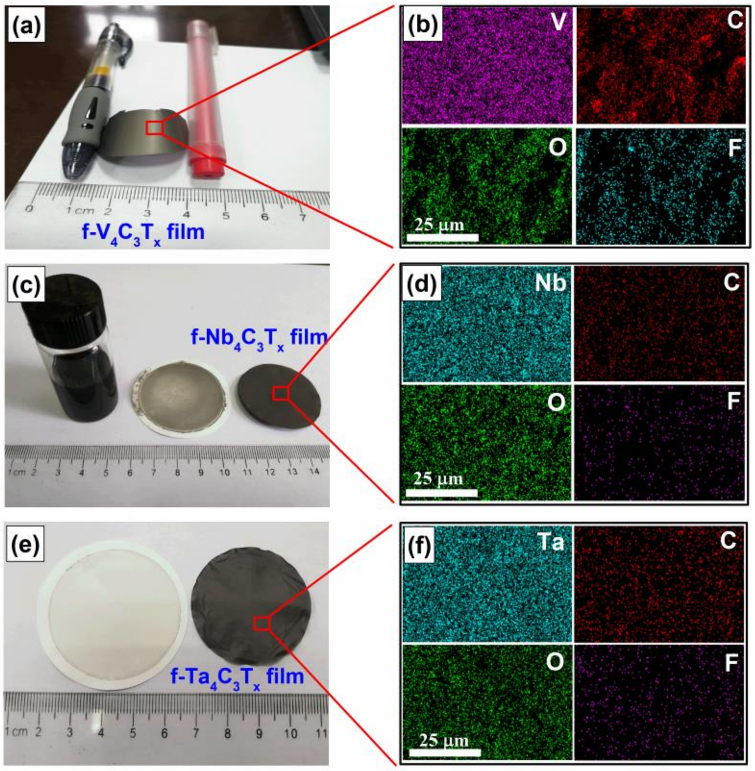

Fig. S14 Photographs and TEM-EDX elemental mapping images of the few-layer $M_4C_3T_x$ (M=V, Nb, Ta) free-standing films: f-$V_4C_3T_x$ **(a-b)**, f-$Nb_4C_3T_x$ **(c-d)**, f-$Ta_4C_3T_x$ **(e-f)**.

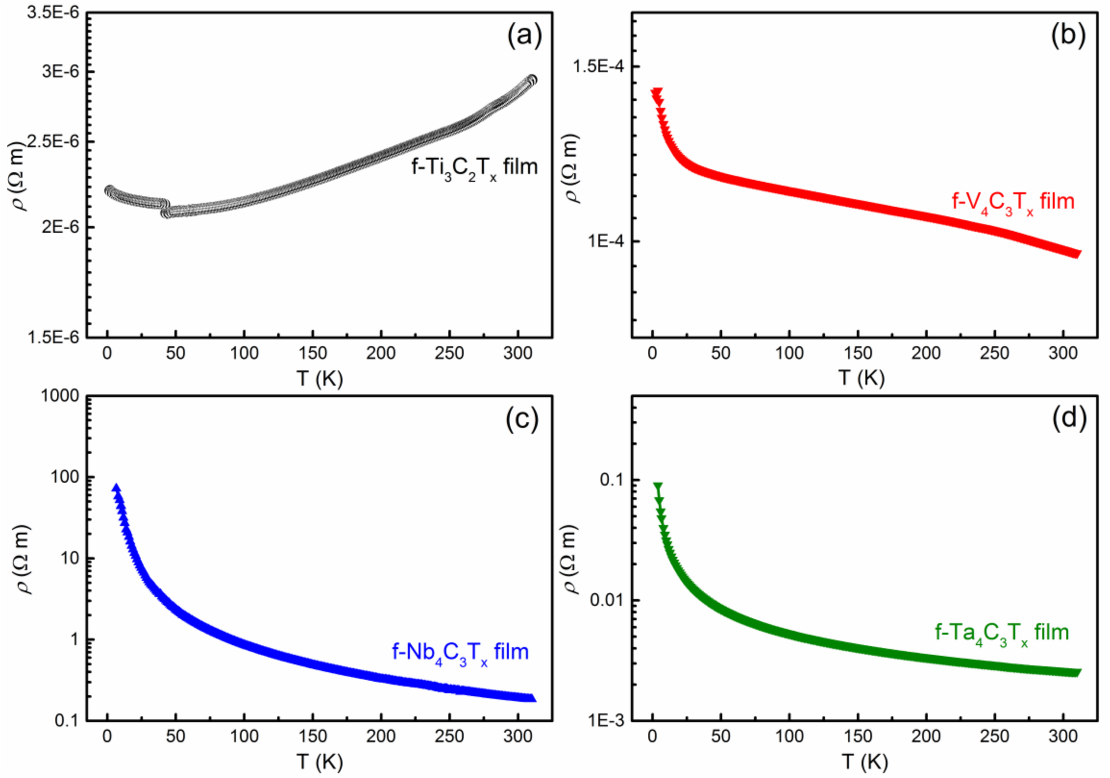

Fig. S15 Temperature dependent resistivity of the few-layer MXene free-standing films: f-$Ti_3C_2T_x$ **(a)**, f-$V_4C_3T_x$ **(b)**, f-$Nb_4C_3T_x$ **(c)**, f-$Ta_4C_3T_x$ **(d)**.

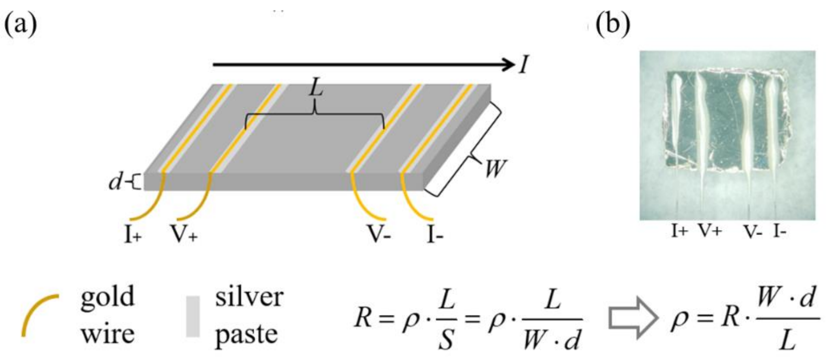

Fig. S16. Schematic diagram **(a)** and photograph of electrodes **(b)** for our free-standing MXene films. The corresponding data processing formula is also given at the bottom.
